# Immunohistochemical Expression of Vitamin D Receptors (VDRs) and Estrogen Receptor Beta 1 (ERβ1) in Molecular Subtypes of Triple-Negative Breast Cancer Tumors: A Cross-Sectional Study

**DOI:** 10.7759/cureus.77637

**Published:** 2025-01-18

**Authors:** Shankaramurthy K N, Basavaraj B Devaranavadagi, Indira A Hundekari

**Affiliations:** 1 Biochemistry, Bijapur Lingayat District Educational (BLDE) Shri B.M. Patil Medical College Hospital and Research Centre, Vijayapura, IND

**Keywords:** breast cancer, erβ1, immunoreactivity, tnbc, vdr

## Abstract

Introduction

Breast cancer (BC) is still the most common malignancy among women globally, and triple-negative breast cancer (TNBC) presents major therapeutic and management issues due to its aggressive nature. Recent studies suggest that the vitamin D receptor (VDR) and estrogen receptor beta 1 (ERβ1) play crucial roles in regulating TNBC progression. Increased expression of VDR and ERβ1 has been linked to tumor suppression, highlighting their potential to impact cancer progression via various signaling pathways. This study analyzes VDR and ERβ1 expressions in TNBC subtypes to discover potential therapeutic targets and improve treatment outcomes for this challenging BC subtype.

Method

This cross-sectional study analyzed 30 invasive ductal carcinoma (IDC) cases of TNBC subtypes using formalin-fixed paraffin embedding (FFPE) tissues. Immunohistochemistry assessed cytoplasmic and nuclear VDR and ERβ1 expression, scoring staining intensity and extent, categorized as negative/low, moderate, or high expression.

Results

High VDR and ERβ1 expressions were analyzed across molecular subtypes of TNBC to explore their therapeutic potential, particularly in TNBC. In TNBC, a high VDR expression was observed in the cytoplasm (n = 10, 33.3%) and the nucleus (n = 2, 6.6%), with statistical significance (p < 0.042). Luminal A cases demonstrated high VDR expression in the cytoplasm (n = 6, 20%) and the nucleus (n = 2, 6.6%) (p < 0.042), while luminal B exhibited high VDR expression exclusively in the cytoplasm (n = 4, 13.3%) (p < 0.042). In HER2-enriched, high VDR expression was confined to the nucleus (n = 3, 10%) (p < 0.042). ERβ1 expression patterns in TNBC showed moderate cytoplasmic expression (n = 9, 50%) and high cytoplasmic expression (n = 1, 5.5%), with statistical significance (p < 0.025). By contrast, luminal A displayed moderate cytoplasmic expression (n = 3, 16.6%) and high cytoplasmic expression (n = 5, 27.7%) (p < 0.025). These findings suggest that VDR and ERβ1 exhibit subtype-specific expression patterns, with significant expression in TNBC, indicating their potential as therapeutic targets.

Conclusion

VDR and ERβ1 expressions differ between TNBC subtypes, indicating their potential as targeted therapies, particularly in TNBC.

## Introduction

Globally, breast cancer (BC) is the most prevalent cancer diagnosed in women and the primary cause of cancer-related mortality [1]. Triple-negative breast cancer (TNBC) is one of the most perilous kinds of BC. The lack of important receptors, including the human epidermal growth factor receptor 2 (HER2), progesterone receptor (PR), and estrogen receptor (ER), makes treatment extremely difficult [2]. About 15% to 20% of all invasive BCs are TNBC, which is linked to a higher risk of distant metastases and early death, usually within three to five years after diagnosis [3]. The majority of TNBC patients are young, premenopausal women. Patients with TNBC still have a poor prognosis despite advances in treatment options such as radiation therapy, chemotherapy, and surgery [4]. Experimental studies suggest that patients with TNBC often have significantly lower serum 25-hydroxyvitamin D (25(OH)D) levels [5]. This potential involvement is further highlighted by findings showing that high vitamin D receptor (VDR) expression correlates with better survival outcomes in TNBC patients [6]. The VDR axis is mainly activated by its binding to DNA, emphasizing the importance of understanding how cytoplasmic VDR expression impacts TNBC phenotype and pathogenesis [7,8].

In addition to VDR, emerging evidence suggests that TNBC progression may also be influenced by estrogen receptor beta (ERβ). ERβ expression is greater in normal breast tissue than in malignant breast tissue. In TNBC, decreased ERβ expression has been linked to increased tumor growth and invasiveness [9,10]. ERβ exhibits non-nuclear signaling functions, highlighting its involvement in non-genomic pathways [11]. BC cells express various ERβ isoforms, including ERβ1, ERβ2, ERβ3, ERβ4, and ERβ5, with only ERβ1 retaining an intact ligand-binding domain, making it a preferred clinical target [2]. Although ERβ1 is primarily localized in the nucleus [12], evidence for its cytoplasmic localization in human breast tumors remains less well-established [13]. In a preclinical mouse model with human TNBC xenografts, increased ERβ1 expression significantly reduced primary tumor growth and metastasis. However, the precise role of ERβ1 in TNBC remains to be fully understood [14].

The present study demonstrated variations in VDR expression across TNBC, luminal A, luminal B, and HER2-enriched, suggesting distinct receptor patterns. The cytoplasmic position of ERβ1 in TNBC and luminal A emphasizes the necessity of tailored treatment approaches for each subtype. The study aims to contribute to finding future novel therapeutic targets by exploring receptor expression patterns, specifically in TNBC, which may lead to novel combination therapy approaches.

## Materials and methods

Study population

The study focused on IDC due to its high prevalence among BC subtypes and its significant clinical relevance. This study used human tissue, and all protocols followed ethical criteria to ensure compliance with regulatory norms. An Institutional Ethical Certificate (approval number: BLDE (DU)/IEC/631-C/2022-2023) dated 28/5/2022 was obtained from the Bijapur Lingayat District Educational (BLDE) (Deemed to be University) Institutional Ethical Committee in Bijapur, India. The tissue samples that were examined were formalin-fixed paraffin embedding (FFPE) tissue blocks that were acquired from the Department of Pathology between August 2018 and May 2023. The study included 30 cases of IDC in female patients aged 30 to 75 years (mean age: 56). The breast cancers were categorized using immunohistochemical analysis based on the expression of hormone receptors (ER and PR) and HER2. The TNBC subtypes were divided into the following groups: TNBC (ER-ve, PR-ve, HER2-ve), luminal A (ER +ve, PR +ve, HER2-ve), and luminal B (ER +ve, PR +ve or -ve, HER2 +ve), HER2-enriched (ER -ve, PR -ve, HER2 +ve) [15]. The distribution of patients in our cross-sectional study was as follows: TNBC (n = 15), luminal A (n = 8), and luminal B (n = 4), HER2-enriched (n = 3). Clinicopathological details, including the patient’s age, hormone receptor and HER2 status, histological data describing the cancer subtype, and grades (I and II), were obtained from hospital records.

Immunohistochemical staining

Tissue sections were cut to a thickness of 4 µm as part of the immunohistochemical staining, and then they were deparaffinized using several xylene changes. Antigen retrieval was performed in EDTA buffer at 120°C for 15 minutes after sections were cooled to room temperature and rinsed with distilled water. They were washed with Tris-buffered saline (TBS) (pH 7.4 to 7.6) and treated with a background blocker from Diagnostic Biosystems (DBS, Pleasanton, CA, USA) for five minutes to reduce nonspecific binding. Mouse monoclonal antibodies targeting VDR at a 1:300 dilution, as previously described (anti-VDR, sc-13133, Santa Cruz Biotechnology, CA, USA) [16] and ERβ1 at a 1:50 dilution (ERβ1-sc-390243, Santa Cruz Biotechnology, CA, USA; dilution range of 1:50-1:500 as per kit literature) were then applied at room temperature for one hour. After thorough washing with TBS, the sections were incubated for 20 minutes with poly horseradish peroxidase (DBS, Pleasanton, CA, USA) for signal amplification. DAB (diaminobenzidine) chromogen was used for five minutes, then counterstaining with Mayer’s hematoxylin. Finally, the sections were dehydrated and mounted with a permanent solution.

VDR and ERβ1 receptor expression

**Figure 1:**
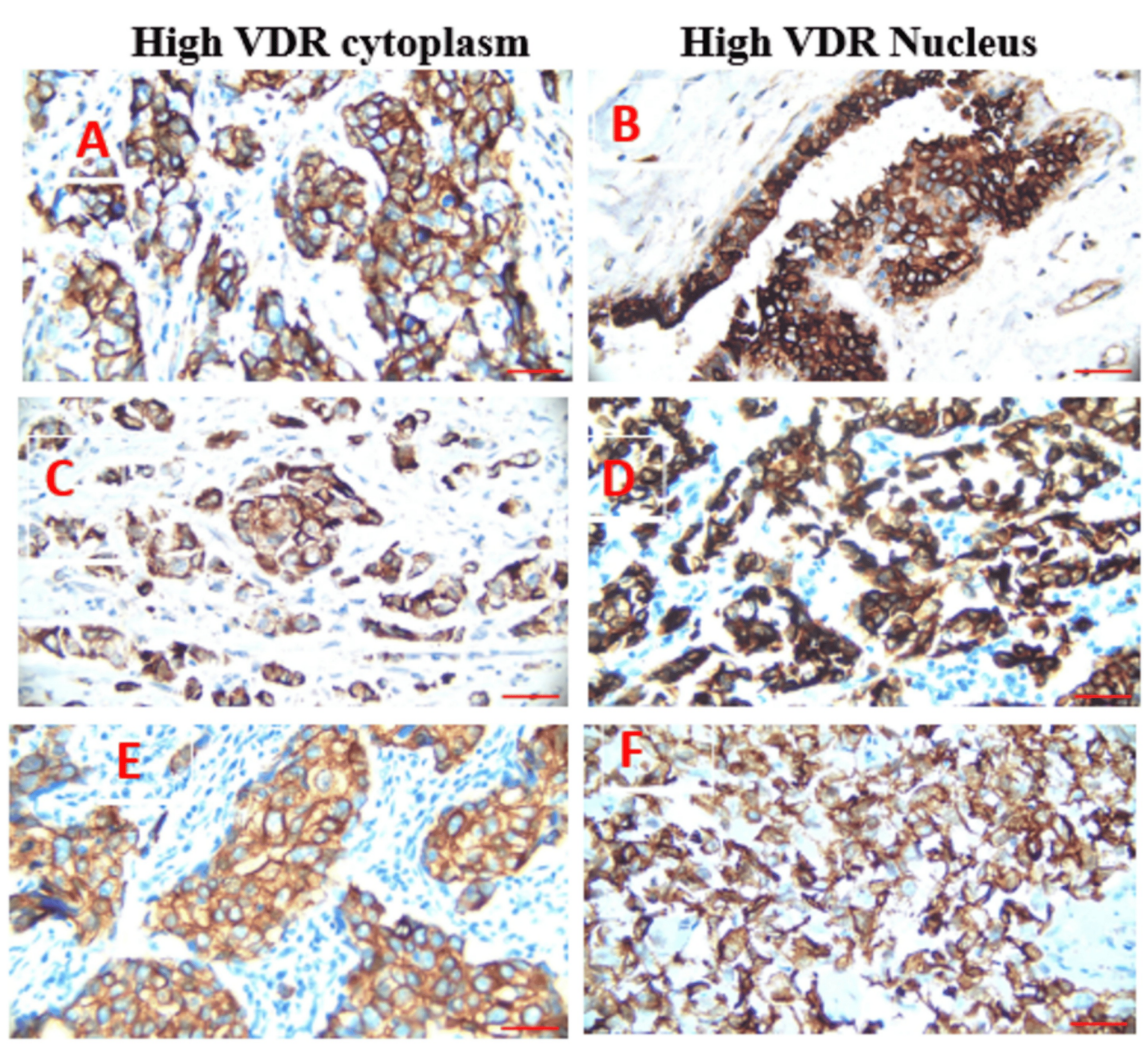
VDR localization (40X) in TNBC subtypes showing high expression in the cytoplasm and nucleus. (A-B) TNBC: (A) High VDR expression in the cytoplasm, (B) high VDR expression in the nucleus. (C-D) Luminal A: (C) High VDR expression in the cytoplasm, (D) high VDR expression in the nucleus. (E) Luminal B: High VDR expression in the cytoplasm. (F) HER2-enriched: High VDR expression in the nucleus. Scale bar = 13.75 µm Abbreviations: VDR, vitamin D receptor; HER2, human epithelial growth factor receptor 2; TNBC, triple-negative breast cancer

The expression of the VDR was evaluated in various subcellular compartments of TNBC subtypes (see Figure 1). VDR staining was observed in both the cytoplasm and the nucleus. The intensity of cytoplasmic staining was categorized into four levels: negative (0), weak (1+), moderate (2+), and strong (3+). VDR staining was considered positive if at least 20% of tumor cells exhibited the highest staining intensity. For nuclear staining, a nucleus was deemed positive if at least 10% of the cells displayed detectable staining [17]. Figure 1 depicts TNBC subtypes with high VDR staining in both the cytoplasm and the nuclear sites.

**Figure 2:**
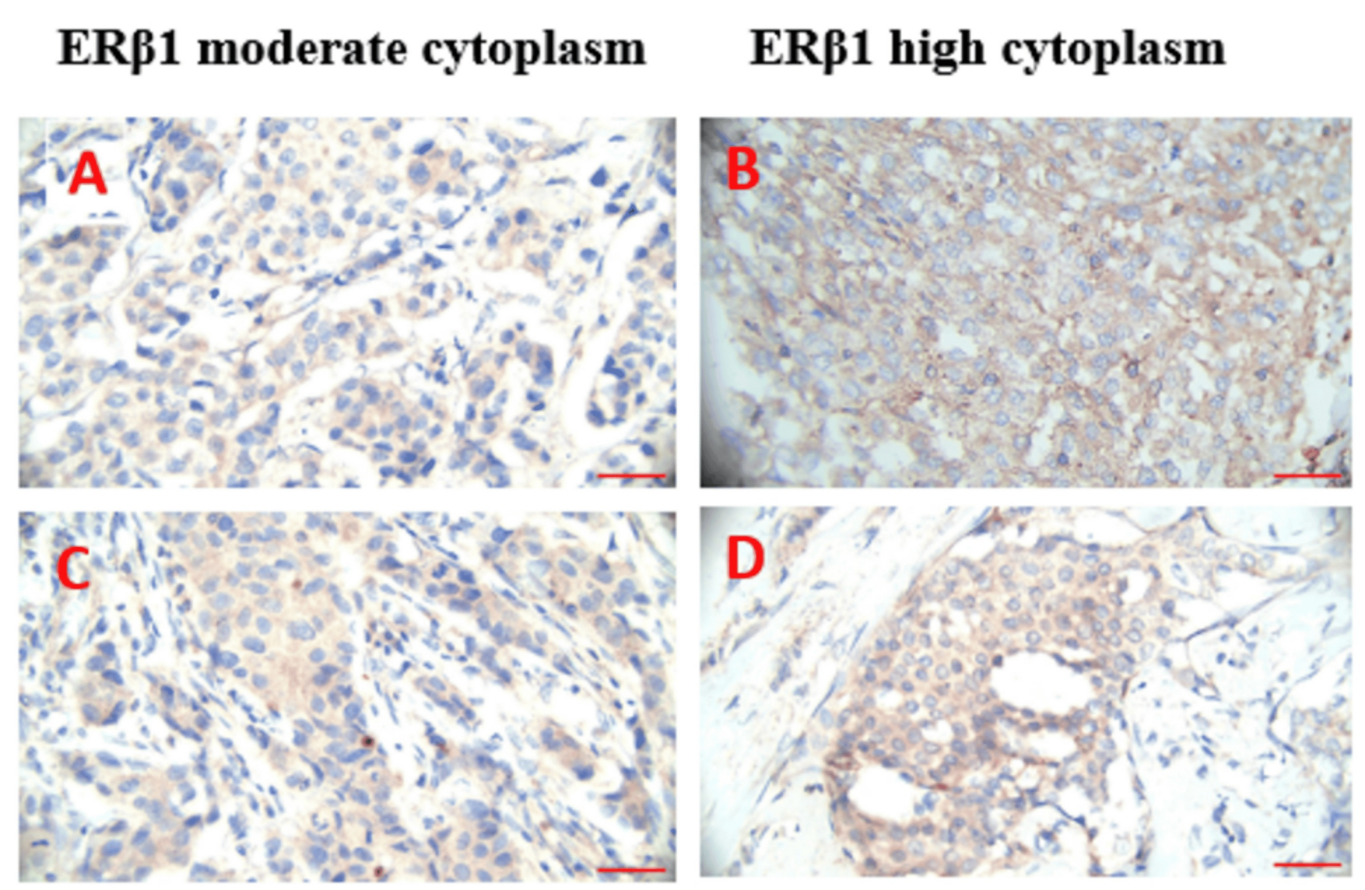
ERβ1 moderate to high expression in the cytoplasm (40X) in TNBC and luminal A. (A-B) TNBC: (A) Moderate ERβ1 expression in the cytoplasm, (B) High ERβ1 expression in the cytoplasm. (C-D) Luminal A: (C) Moderate ERβ1 expression in the cytoplasm, (D) High ERβ1 expression in the cytoplasm. Scale bar: 13.75 µm Abbreviations: ERβ1, estrogen receptor beta 1; TNBC, triple-negative breast cancer

To assess ERβ1 expression in TNBC and luminal A tumor cells, a scoring system that evaluated both the extent and intensity of staining was utilized [18]. ERβ1 immunoreactivity was considered positive if more than 10% of the cells showed cytoplasmic staining [11]. The extent of staining was categorized as follows: 0 for < 1% positive cells, 1 (1%-25%), 2 (26%-50%), 3 (51%-75%), and 4 (76%-100%). Intensity was scored on a scale from negative (0), weak (1+), moderate (2+), and strong (3+). The combined scores were then grouped into three categories: ERβ1-negative/low (0-2), moderate (3-5), and high (6-7) [18]. This study presents the percentage of tumors exhibiting only cytoplasmic staining, with a representative tumor showing moderate to high staining intensity and cytoplasmic localization, as shown in Figure 2. A pathologist examined the immunohistochemically stained slides using light microscopy (LABOMED, Lx500, Hicksville, New York). Images were captured with a microscope slide scanner (Accident Reconstruction Professional (AR Pro) software, Dirigo Software, LLC, USA).

Statistical analysis

Categorical data were presented as frequencies and percentages. The Chi-square test was used to analyze associations between variables. This test helped determine whether there were significant differences in the expression of VDR and ERβ1 among various TNBC subtypes, including TNBC, luminal A, luminal B, and HER2-enriched. A p-value of <0.05 was considered statistically significant for all comparisons. All data analysis was conducted using IBM SPSS Statistics for Windows, Version 28.0 (released 2021, IBM Corp., Armonk, NY) to ensure a comprehensive statistical examination of the results.

## Results

Patterns of VDR expression in TNBC subtypes

The analysis of VDR expression, including TNBC, luminal A, luminal B, and HER2-enriched, focused on cytoplasmic and nuclear localization. Significant differences in VDR localization patterns were observed across subtypes. In TNBC, high VDR expression was predominantly cytoplasmic (n = 10, 33.3%), with nuclear expression detected in a smaller subset (n = 5, 16.6%) (p < 0.042). This dual localization pattern suggests a distinct functional role for VDR in TNBC. For luminal A, high cytoplasmic VDR expression was observed in n = 6 (20%), while nuclear expression was limited to n = 2 (6.6%) (p < 0.042), indicating a more restricted localization compared to TNBC. In luminal B, high VDR expression was observed exclusively in the cytoplasm (n = 4, 13.3%), with no nuclear expression detected (p < 0.042). In HER2-enriched, no cytoplasmic expression was observed, but nuclear VDR localization was identified in n = 3 (10%) (p < 0.042), suggesting a unique expression profile for this subtype. Table 1 summarizes these patterns, highlighting the significant variability in VDR expression and localization across subtypes. Representative images of VDR staining for each subtype are illustrated in Figure 1. TNBC displayed a high cytoplasmic VDR expression. However, differences in nuclear localization were less pronounced among subtypes.

**Table 1:** Expression of VDR in tumor cells in molecular subtypes of breast cancer * Indicates statistical significance (p < 0.05). Abbreviations: VDR, vitamin D receptor; TNBC, triple-negative breast cancer; HER2, human epithelial growth factor receptor 2

Staining intensity	VDR expression in cellular location	TNBC, n = 15	Luminal A, n = 8	Luminal B, n = 4	HER2-enriched, n = 3	p-value
High	VDR cytoplasm, n (%)	10 (33.3)	6 (20)	4 (13.3)	0 (0)	p<0.042*
	VDR nucleus, n (%)	5 (16.6)	2 (6.6)	0 (0)	3 (10)	

Cytoplasmic ERβ1 immunoreactivity in TNBC and luminal A

Immunohistochemical analysis revealed ERβ1 immunoreactivity exclusively in the cytoplasm of tumor cells in both TNBC and luminal A subtypes, with no nuclear staining detected. This study examined ERβ1 expression in 18 IDC cases, focusing specifically on TNBC (n = 10) and luminal A (n = 8). Other subtypes, including luminal B and HER2-enriched, were excluded due to limited tissue availability. ERβ1 expression was evaluated based on cytoplasmic staining intensity. In TNBC, moderate cytoplasmic ERβ1 expression was observed in n = 9 (50%), while high expression was detected in n = 1 (5.5%) (p < 0.025). In contrast, luminal A displayed moderate expression in n = 3 (16.6%) and high expression in n = 5 (27.7%) (p < 0.025). These findings are summarized in Table 2 and illustrated in Figure 2. TNBC has considerably greater moderate cytoplasmic ERβ1 expression than luminal A. These findings highlight the importance of ERβ1 expression in identifying variation between TNBC and luminal A.

**Table 2:** Expression of ERβ1 in TNBC and luminal A * Indicates statistical significance (p < 0.05). Abbreviations: ERβ1, estrogen receptor beta 1; TNBC, triple-negative breast cancer

Staining intensity	ERβ1 expression in cellular location	TNBC, n = 10	Luminal A, n = 8	p-value
ERβ1 cytoplasm	Moderate, n (%)	9 (50)	3 (16.6)	p<0.025*
	High, n (%)	1 (5.5)	5 (27.7)	

## Discussion

This cross-sectional analysis highlights important VDR and ERβ1 expression patterns in TNBC subtypes. Our analysis showed that TNBC VDR was significantly expressed in the cytoplasm in 33.3% and the nucleus in 16.6%. Remarkably, TNBC exhibited no moderate nuclear expression and very little moderate cytoplasmic expression. In line with recent research, TNBC tumors exhibited low nuclear VDR expression, with 56.6% being VDR-negative [17]. The findings that lower VDR expression is linked to a worse prognosis in TNBC, along with the inverse relationship between VDR expression and tumor aggressiveness, suggest that VDR may be a key factor in determining outcomes in this subtype [19]. Our study also showed that VDR expression in luminal A exhibited high cytoplasmic staining in 20% and nuclear expression in 6.6%. However, by contrast, a study by Huss et al. found that 6.6% of luminal A-like tumors showed negative VDR expression in the nucleus [16]. In the current study, 13.3% of luminal B showed cytoplasmic VDR expression; however, no discernible nuclear staining was found. This outcome is in line with previous studies by Huss et al. who found that 25.6% of luminal B tumors had nuclear VDR expression [16]. Notably, HER2-enriched tumors in the present study showed a distinct expression pattern, with 10% expressing nuclear VDR and no cytoplasmic VDR. Nevertheless, prior research has demonstrated a connection between VDR expression and HER2 status, with cytoplasmic VDR expression being much higher in HER2-enriched tumors [16]. These results highlight the need for a more thorough understanding of the role of VDR in different TNBC subtypes, which could have consequences for future therapies that target this receptor.

Our findings show unique ERβ1 expression profiles in TNBC and luminal A cases. In TNBC, 50% exhibited moderate cytoplasmic ERβ1 expression, while 5.5% showed high expression. This suggests that ERβ1 may play a role in non-genomic signaling pathways relevant to this aggressive subtype. According to Reese et al., cytoplasmic ERβ1 expression was present in BC at a significantly higher frequency, with high cytoplasmic expression in 70.1% of TNBC cases [18]. Additionally, it has been observed that cytoplasmic ER immunoreactivity is present in 23% of ER-negative BC cases, while it is present in 1.4% of ER-positive BC cases [11]. These results highlight ERβ1 special function in TNBC, especially its cytoplasmic location, which may have unique functional consequences for tumor and treatment response. According to our findings, ERβ1 expression was significantly lower in luminal A compared to TNBC, with moderate cytoplasmic expression seen in 16.6% and high cytoplasmic expression in 27.7%. Similar to TNBC, luminal A lacked nuclear ERβ1 expression, indicating that ERβ1 functional role in genomic signaling is restricted in these subtypes. Reese et al. reported low or negative cytoplasmic ERβ1 expression in 25.5% of cases, with a rare occurrence of high nuclear expression, consistent with the primarily low expression profile [18]. The receptor is implicated in pathways that regulate tumor development and survival due to its uneven expression. In addition, it suggests that the aggressive characteristics of TNBC are linked to VDR and ERβ1 expression.

Strength and limitations

Our primary focus is on the unique subcellular location of VDR and ERβ1 in TNBC subtypes. It highlights the significance of cytoplasmic receptor distribution and offers a fresh viewpoint on how it may impact tumor growth and responsiveness to treatment. However, the reliability of the results is limited by the small sample size. The study scope is further limited by the exclusion of luminal B and HER2-enriched subtypes from ERβ1 analysis due to tissue availability issues. Moreover, the study's cross-sectional design makes it challenging to demonstrate any direct or temporal relationship between receptor expression and disease outcomes.

## Conclusions

Our study concludes that there are notable variations in VDR and ERβ1 expressions among TNBC subtypes. The predominant cytoplasmic VDR expression in TNBC, coupled with limited nuclear localization, suggests a distinct functional role of VDR in this aggressive subtype. Furthermore, the lack of nuclear staining and the exclusive cytoplasmic ERβ1 expression in both TNBC and luminal A tumors suggest possible variations in receptor signaling and regulatory mechanisms. These findings emphasize the importance of further research into the subtype-specific roles of VDR and ERβ1, which could uncover new therapeutic opportunities for improving TNBC treatment.

Future studies should examine the efficacy of co-targeting VDR and ERβ1, as this could offer significant insights into the prospective benefits of combination therapies. Furthermore, these results need to be confirmed in various demographic groups to highlight the importance of conducting larger multicenter clinical trials.
